# Supplementary figures and images for: Abundance of Oligoflexales bacteria is associated with algal symbiont density, independent of thermal stress in Aiptasia anemones
Source: Ecol Evol. 2023 Dec 6;13(12):e10805. doi: 10.1002/ece3.10805 (PMC10701089; doi:10.1002/ece3.10805)

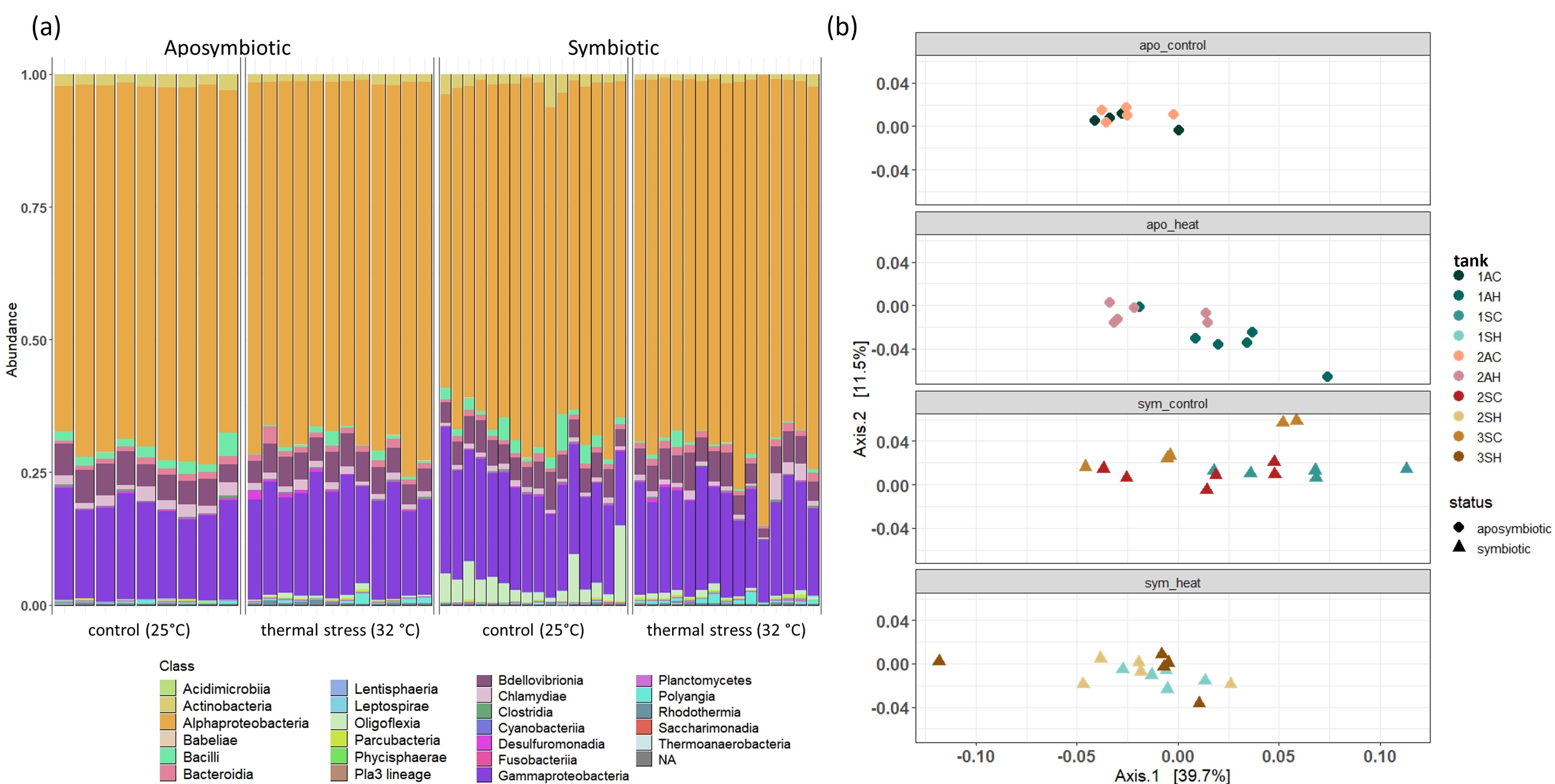

Supplement: Supplementary file 1 — Figure S1 [file ECE3-13-e10805-s007.jpeg]

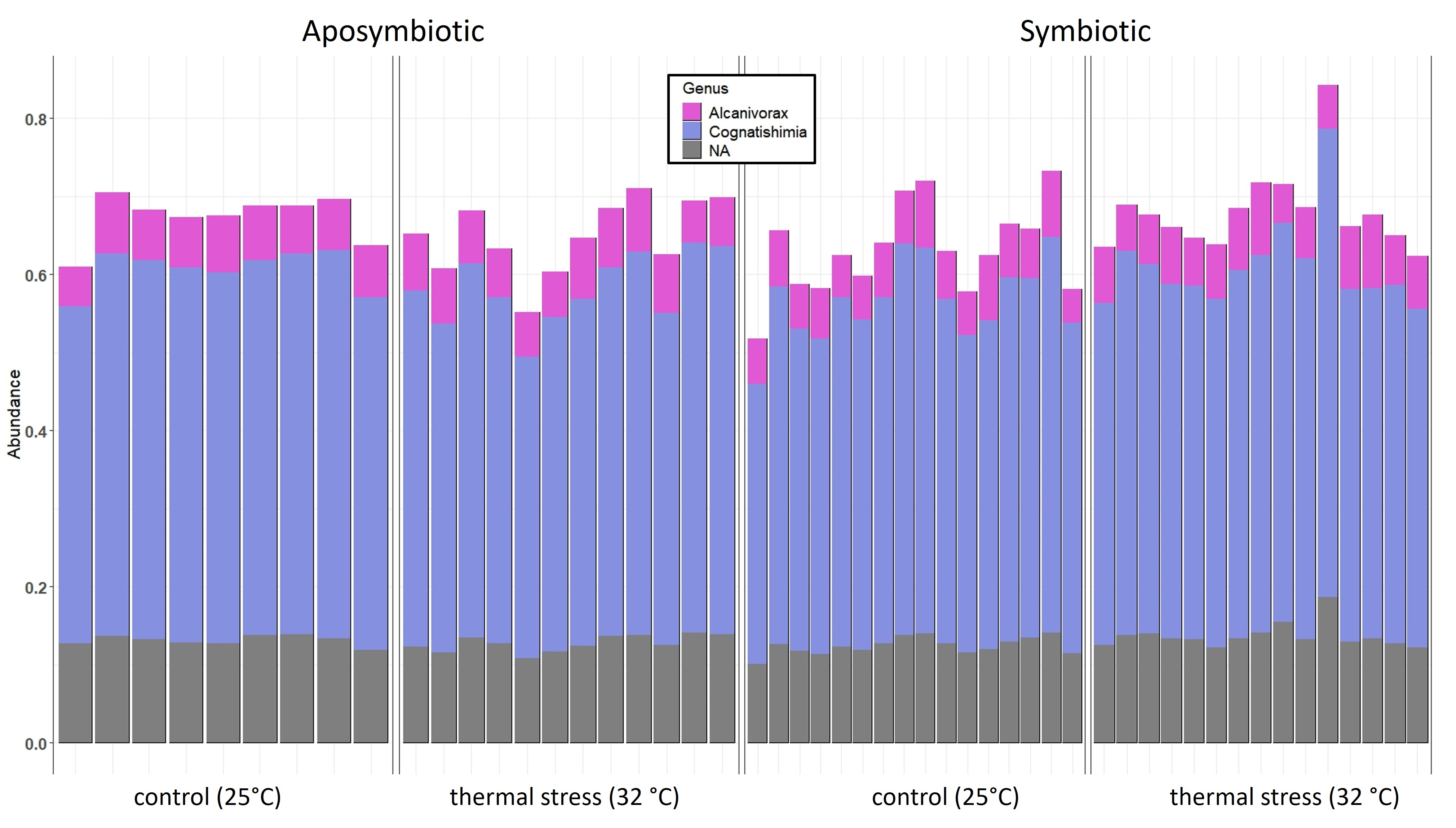

Supplement: Supplementary file 2 — Figure S2 [file ECE3-13-e10805-s006.png]

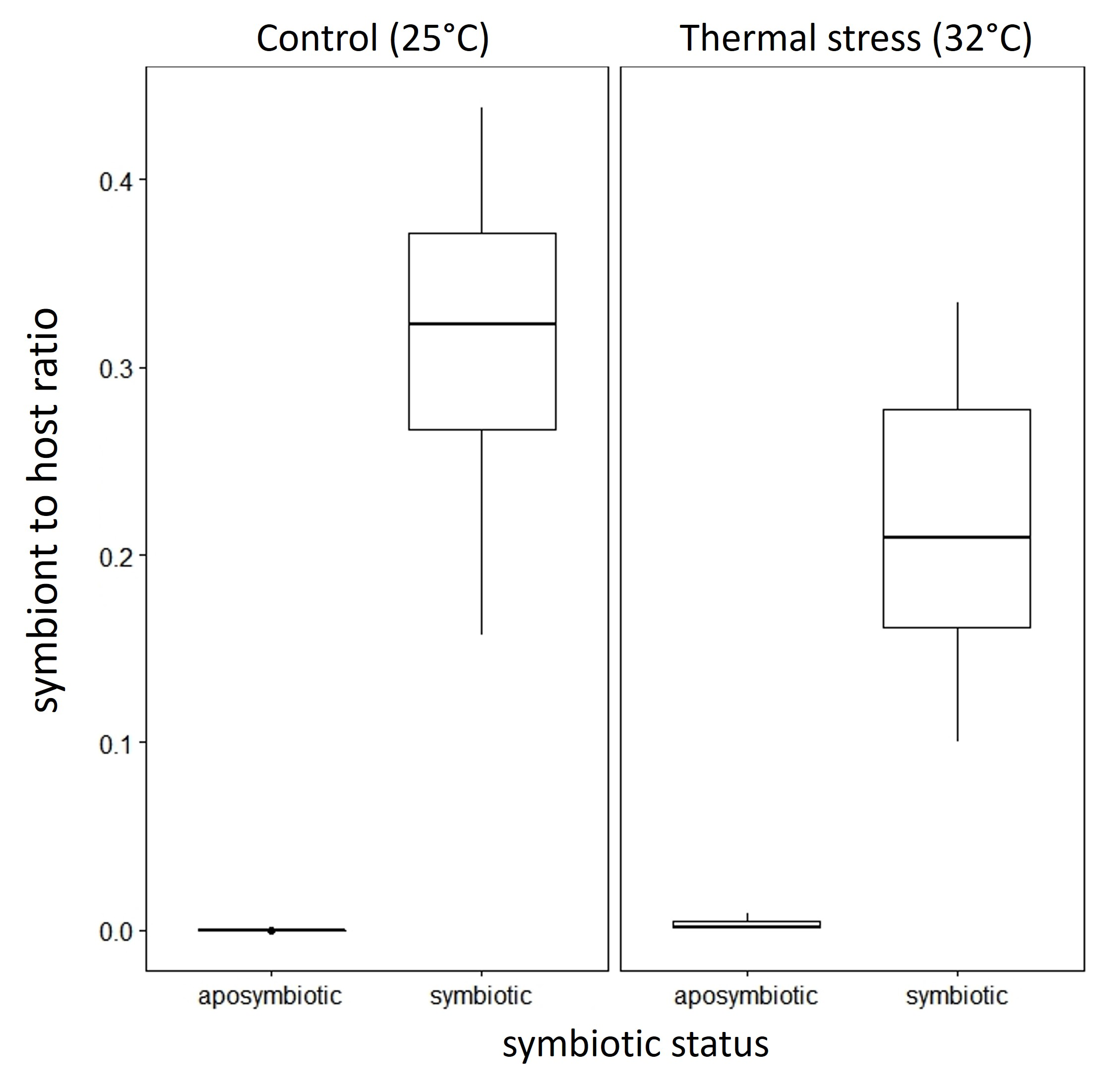

Supplement: Supplementary file 3 — Figure S3 [file ECE3-13-e10805-s001.png]

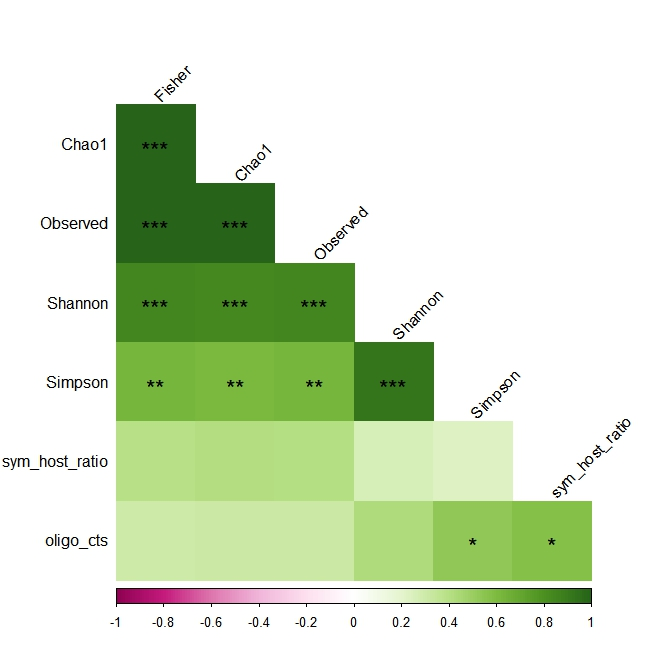

Supplement: Supplementary file 4 — Figure S4 [file ECE3-13-e10805-s002.png]

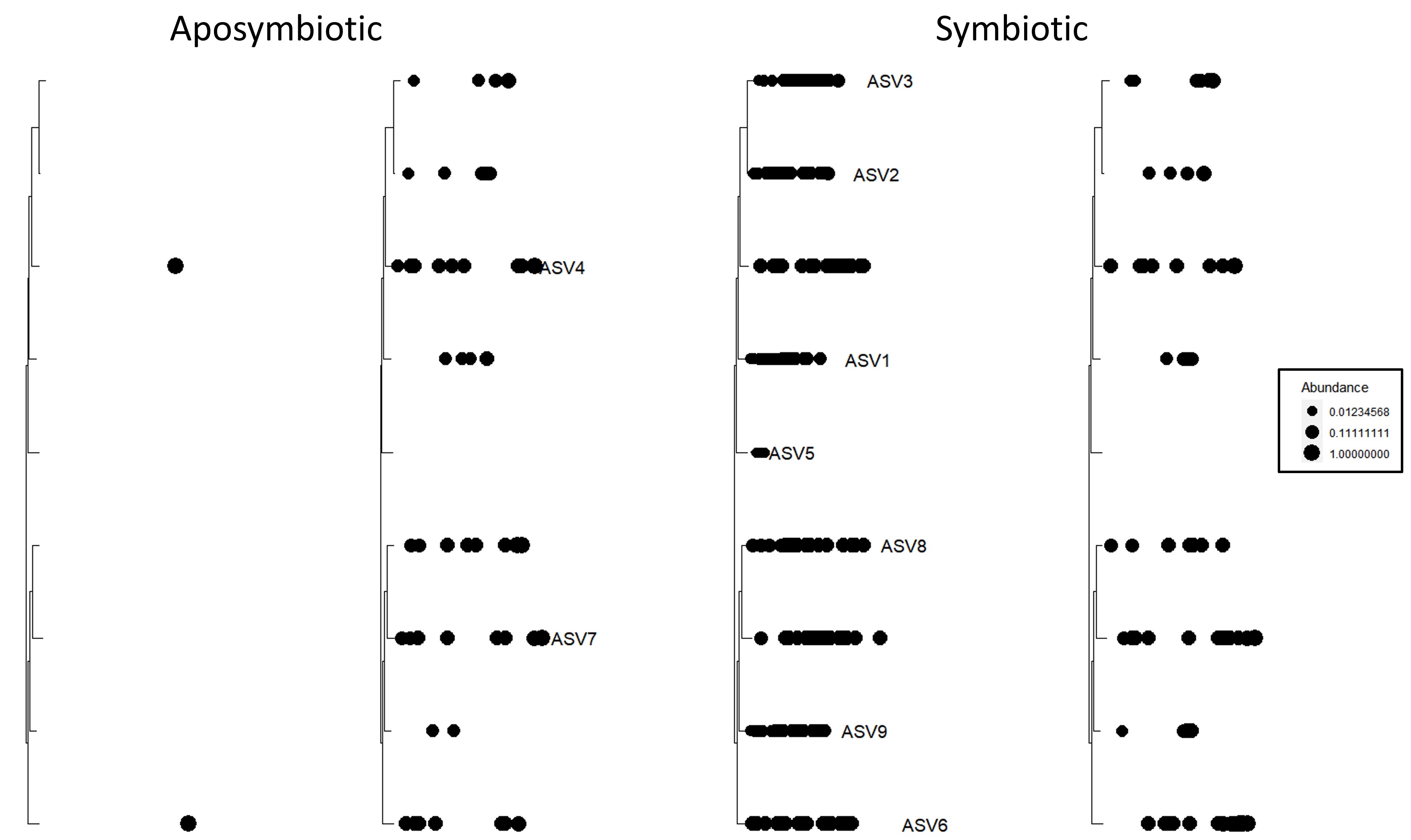

Supplement: Supplementary file 5 — Figure S5 [file ECE3-13-e10805-s004.jpeg]

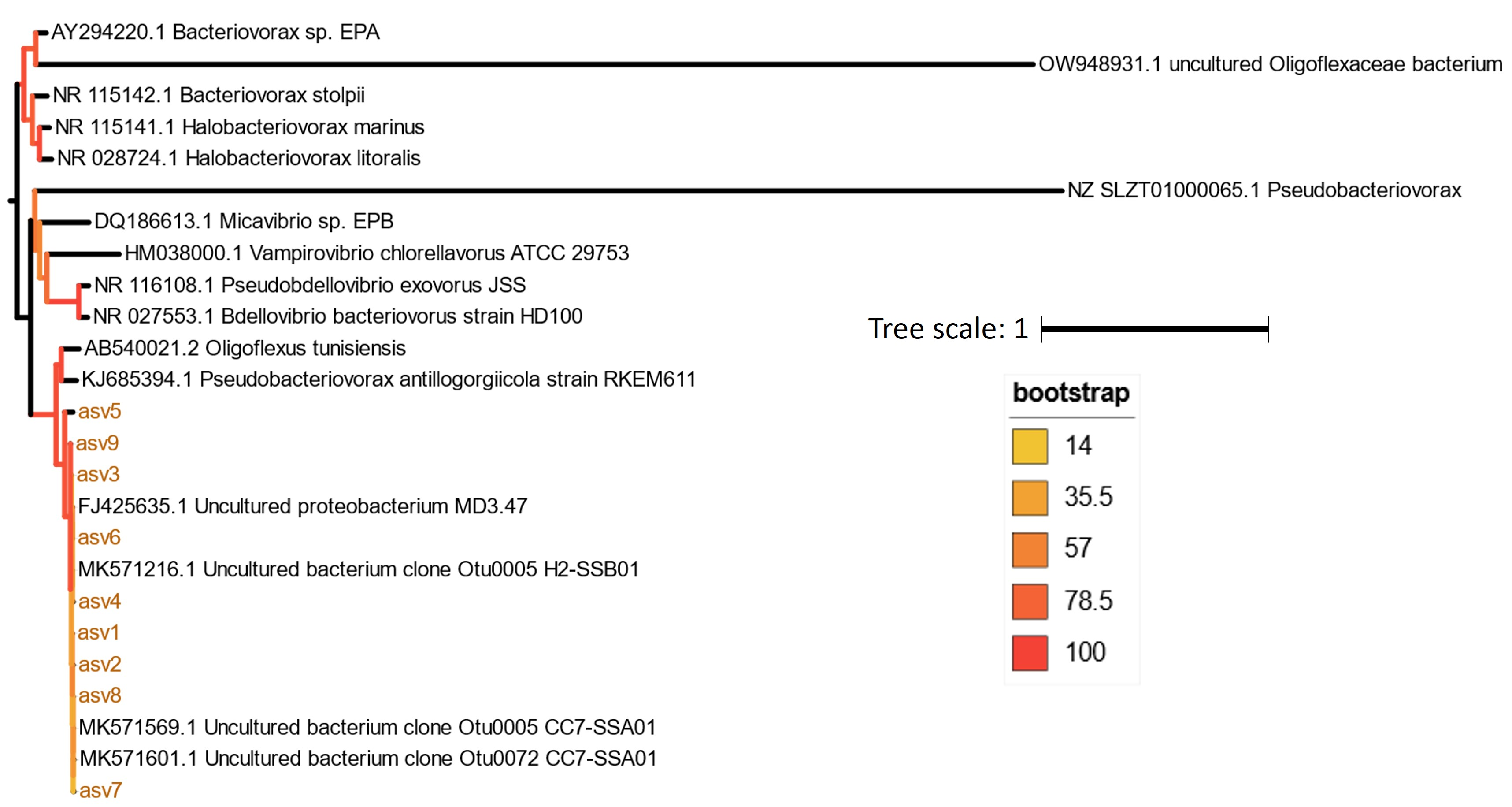

Supplement: Supplementary file 6 — Figure S6 [file ECE3-13-e10805-s003.png]
