# Supplementary material for: Abundance of Oligoflexales bacteria is associated with algal symbiont density, independent of thermal stress in Aiptasia anemones
Source: Ecol Evol. 2023 Dec 6;13(12):e10805. doi: 10.1002/ece3.10805 (PMC10701089; doi:10.1002/ece3.10805)

APPENDIX

Figures A1-A6

Tables A1-Table A8

APPENDIX- FIGURE LEGENDS

Figure A1. (a) Relative abundance barplots of taxa, by class, in aposymbiotic and symbiotic anemones by experimental conditions (25 °C vs 32 °C). Alphaproteobacteria, Bdellovibrionota and Gammaproteobacteria dominate all the samples. Oligoflexia are distinctly present in control symbiotic anemones. (b) Beta differences by weighted Unifrac, principal coordinates of analysis (PCoA) of aposymbiotic anemones (circles, tanks:1AC, 1AH, 2AC and 2AH) and symbiotic anemones (triangles, tanks: 1SC, 1SH, 2SC, 2SH, 3SC and 3SH).

Figure A2. Relative abundance of the three most abundant genera in all Aiptasia groups: *Alcanivorax* belonging to the Oceanospirillales order, *Cognatishimia* and an unclassified bacterium, both genera are part of the Rhodobacterales order.

Figure A3. Symbiont to host ratio (S/H) in Aiptasia anemones. Ribosomal protein L10 was used as a reference for Aiptasia and actin locus gene for *Symbiodinium*. In both treatments, aposymbiotic Aiptasia approximated zero but ratios in symbiotic Aiptasia decreased after mild thermal stress (p= 0.047).

Figure A4. Correlation matrix showing the strength of interactions between S/H ratio (“sym_host_ratio”) and a set of variables containing six alpha diversity indices (Fisher, Chao1, Shannon, Simpson) and Oligoflexales (oligo_cts) counts. S/H ratio displays a significant correlation with one variable, Oligoflexales abundance.

Figure A5. Phylogenetic visualization of Oligoflexales ASV abundance per Aiptasia treatment group (aposymbiotic control, aposymbiotic thermal stress, symbiotic control, and symbiotic thermal stress) using rarefied and filtered data. A total of 9 ASVs were observed. ASV 4 and ASV 6 were observed in all samples but differed in abundance. ASV abundance and diversification in aposymbiotic anemones increased in heat stressed anemones but decreased in symbiotic anemones.

Figure A6. Oligoflexales phylogenetic analysis. Maximum likelihood phylogeny with ultrafast bootstrap (n=1,000 replicates) of this study’s (golden color) Oligoflexales ASVs with two published Oligoflexales sequences (AB540021.2 and OW948931.1), along with four sequences that were close matches to our ASV sequences (FJ425635.1, MK571216.1, MK571569.1, MK571601.1) and members of the Bdellovibrionota phylum (*Bdellovibrionia* and *Bacterovoracia*). Surprisingly, an uncultured Oligoflexaceae bacterium (OW948931.1) did not cluster with the other confirmed member of Oligoflexiales (AB540021.2, *Oligoflexus tunisiensis*) or presumptive members of Oligoflexiales (ASVs in this study) but clustered with a *Bacteriovorax* sp.

APPENDIX- TABLES

Table A1. Read/ASV pipeline using DADA2 and Phyloseq.
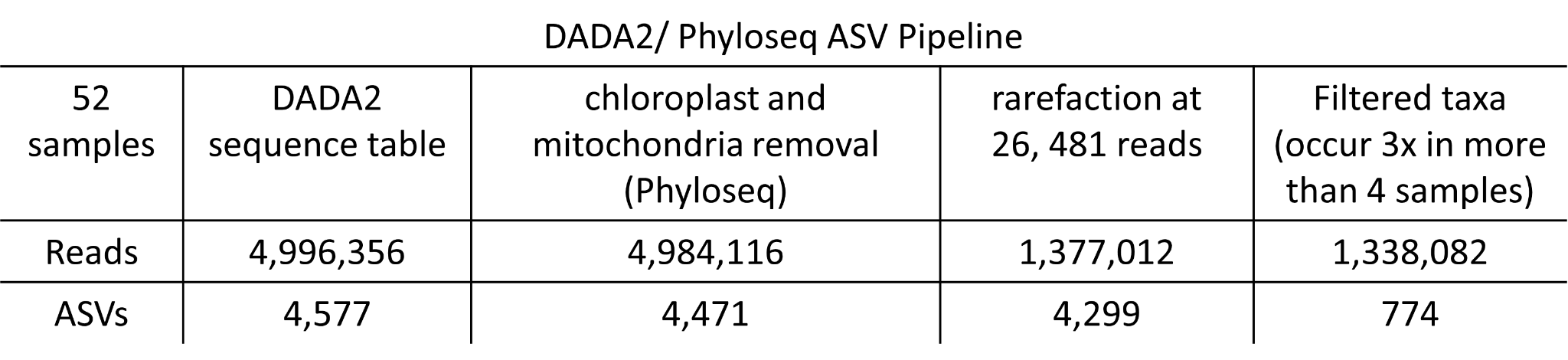


Table A2. Significant, consistent matches to our 9 queried Oligoflexales ASVs, using standard NCBI BLAST (blastn suite). GenBank MK571601.1, MK571569.1 and MK571216.1 originate from (Randle et al. 2020). GenBank FJ425635 derives from an unpublished study on the microbiome of the coral *Orbicella* (formerly *Montastrea) faveolata*.


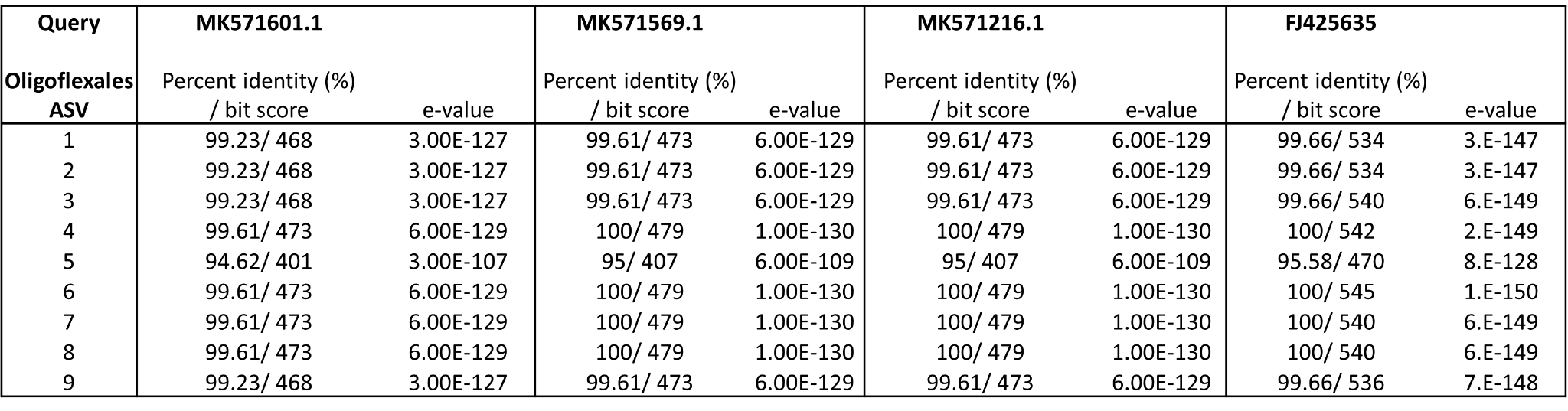


Table A3. Aiptasia samples with sufficient DNA for qPCR analysis to determine S/H ratio. After qPCR analysis, three “symbiotic” samples were omitted from downstream analysis due to incompatibility with the 16S rDNA dataset (these three samples produced low Illumina read yields and were filtered from the sequencing dataset).

**
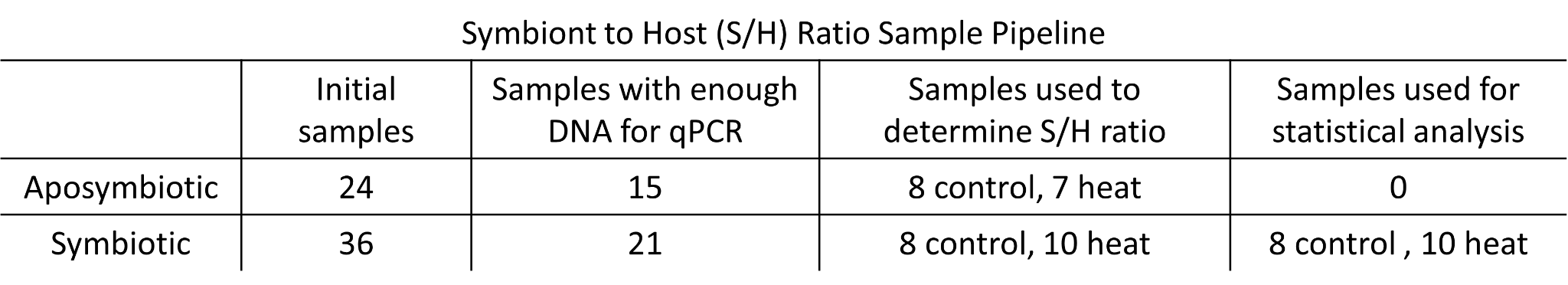
**

Table A4. Average cycle threshold (Ct) values of samples used for qPCR analysis. Primers L10 (host, Aiptasia) and actin (symbiont, *S. linucheae*) were used to calculate S/H ratio using the formula: $( 2^{(Ct host/Ct sym)} ) * 2$
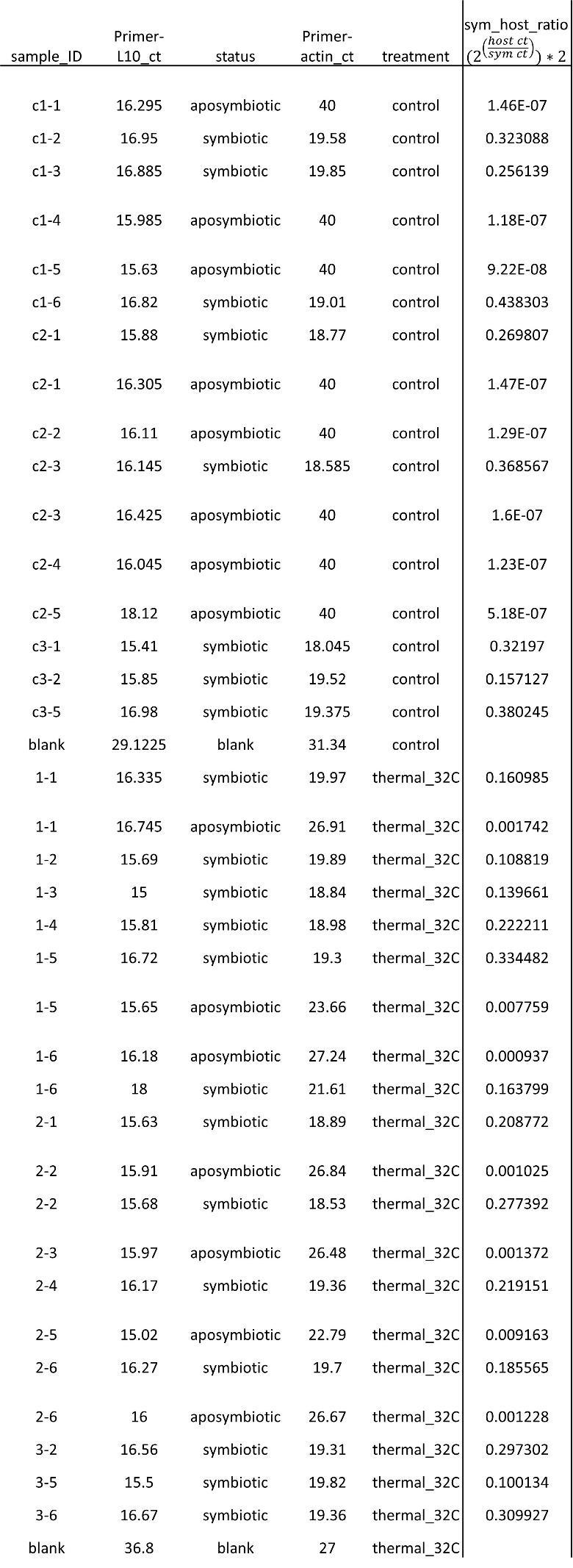


Table A5. Alpha diversity statistical output. A one-way ANOVA test was used to determine alpha diversity (Chao1 index) differences between groups. Post hoc analysis was performed, using Tukey multiple comparison of means (95% family-wise confidence level), to assess which groups are different from the rest.


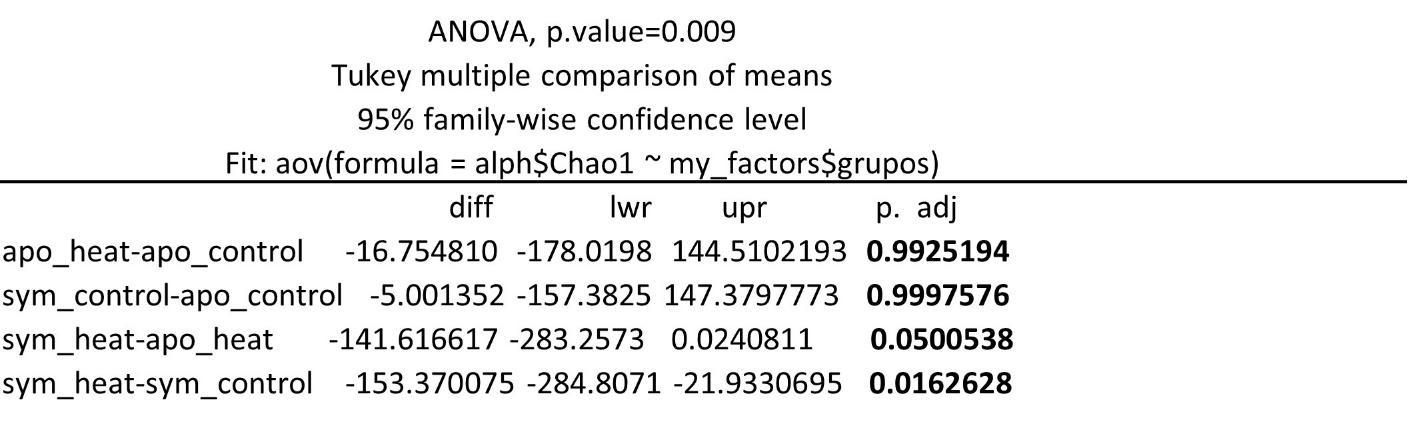


Table A6. Beta diversity statistical output using either the nonparametric test, PERMANOVA for assessing differences between Aiptasia microbial assemblages (symbiotic control vs symbiotic heat stressed, aposymbiotic control vs aposymbiotic heat stressed) with even group dispersions or ANOSIM for microbial assemblages with uneven dispersion (symbiotic control vs aposymbiotic control).


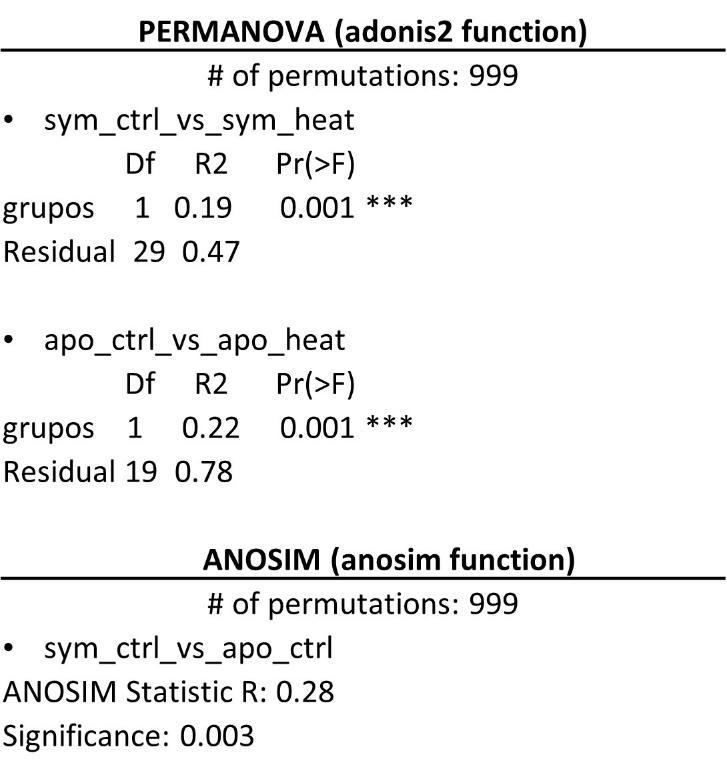


Table A7. Statistical output of linear mixed effects model, fit by residual maximum likelihood (REML), to account for random effects (from tank differences between treatment groups).


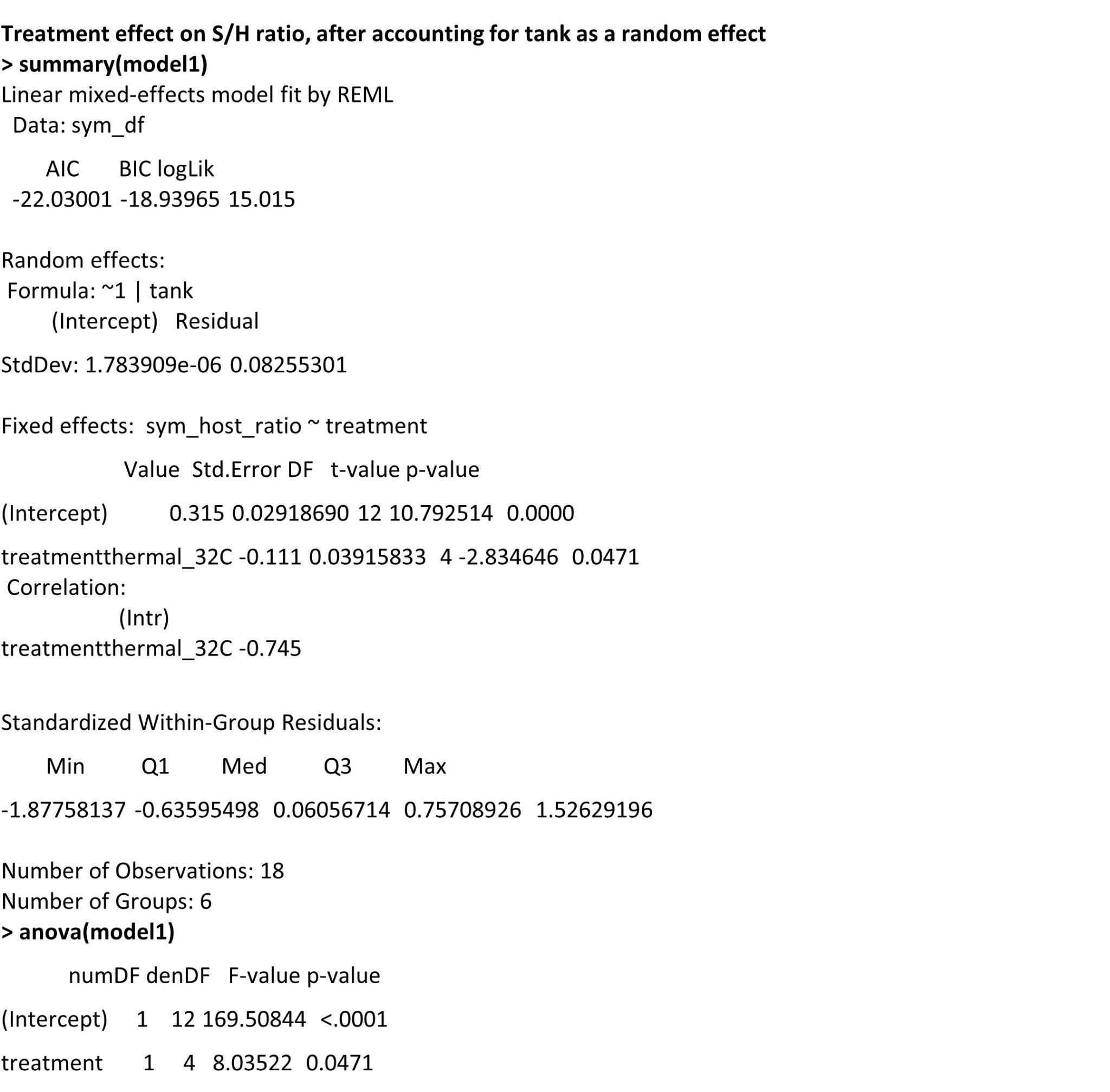


Table A8. Pearson’s correlation p-values, corresponding to the correlation matrix on Fig. A6.
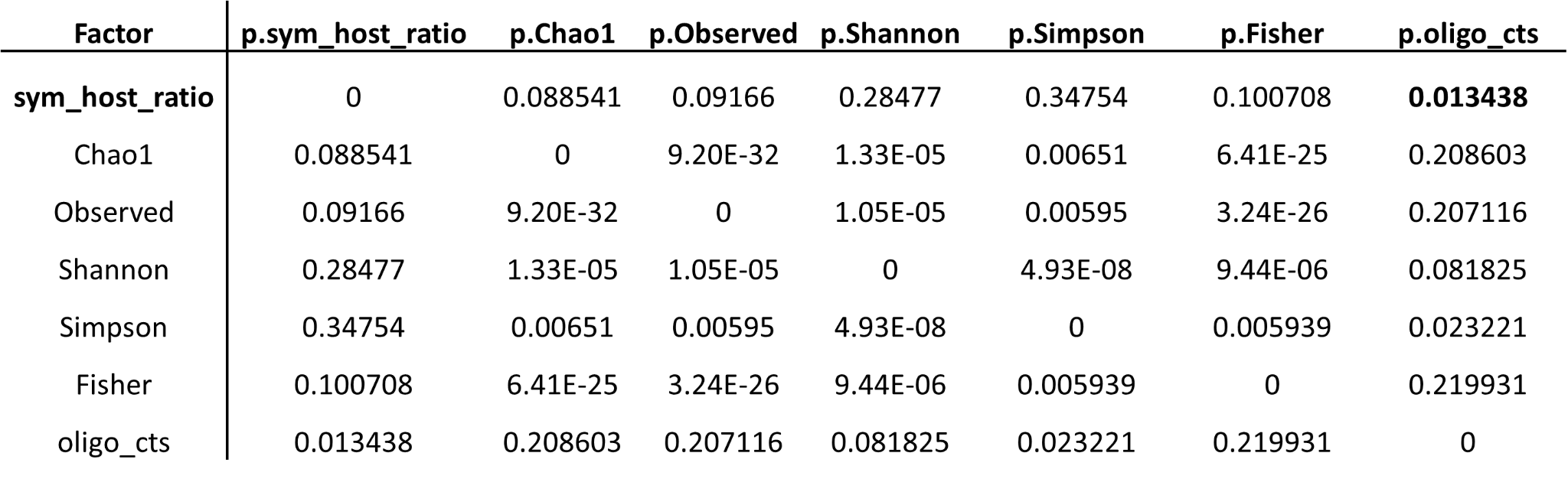

Supplement: Supplementary file 7 — Tables S1–S8 [file ECE3-13-e10805-s005.docx]
